# The Role of Heat Shock Factor 1 in Preserving Proteomic Integrity During Copper-Induced Cellular Toxicity

**DOI:** 10.3390/ijms252111657

**Published:** 2024-10-30

**Authors:** Shruti Ghai, Rejina Shrestha, Ahmad Hegazi, Vanessa Boualoy, Shi-He Liu, Kuo-Hui Su

**Affiliations:** Department of Cell and Cancer Biology, College of Medicine and Life Sciences, The University of Toledo, Toledo, OH 43614, USA; shrutirakesh.ghai@rockets.utoledo.edu (S.G.); rejina.shrestha@rockets.utoledo.edu (R.S.); ahmad.hegazi@rockets.utoledo.edu (A.H.); vanessa.boualoy@rockets.utoledo.edu (V.B.); shi-he.liu@utoledo.edu (S.-H.L.)

**Keywords:** copper toxicity, heat shock factor 1, dihydrolipoamide S-acetyltransferase, cuproptosis, proteostasis

## Abstract

Copper is crucial for many physiological processes across mammalian cells, including energy metabolism, neurotransmitter synthesis, and antioxidant defense mechanisms. However, excessive copper levels can lead to cellular toxicity and “cuproptosis”, a form of programmed cell death characterized by the accumulation of copper within mitochondria. Tumor cells are less sensitive to this toxicity than normal cells, the mechanism for which remains unclear. We address this important issue by exploring the role of heat shock factor 1 (HSF1), a transcription factor that is highly expressed across several types of cancer and has a crucial role in tumor survival, in protecting against copper-mediated cytotoxicity. Using pancreatic ductal adenocarcinoma cells, we show that excessive copper triggers a proteotoxic stress response (PSR), activating HSF1 and that overexpressing HSF1 diminishes intracellular copper accumulation and prevents excessive copper-induced cell death and amyloid fibrils formation, highlighting HSF1′s role in preserving proteasomal integrity. Copper treatment decreases the lipoylation of dihydrolipoamide S-acetyltransferase (DLAT), an enzyme necessary for cuproptosis, induces DLAT oligomerization, and induces insoluble DLAT formation, which is suppressed by overexpressing HSF1, in addition to enhancing the interaction between HSF1 and DLAT. Our findings uncover how HSF1 protects against copper-induced damage in cancer cells and thus represents a novel therapeutic target for enhancing copper-mediated cancer cell death.

## 1. Introduction

Copper is an essential trace element upon which numerous proteins and enzymes are dependent for their participation in critical cellular functions [1,2]. For instance, cytochrome c oxidase, a copper-dependent enzyme integral to cellular respiration, facilitates the generation of adenosine triphosphate (ATP) in mitochondria, which is essential for energy production [3]. Copper also plays a crucial role in converting non-absorbable ferric iron into the absorbable ferrous form, enhancing the efficiency of dietary iron absorption by the small intestine [4]. Moreover, copper contributes significantly to the proper activation of immune cells, thereby bolstering defense against infections and diseases [5]. However, excessive copper accumulation can lead to cellular toxicity and trigger “cuproptosis”, a copper-dependent form of programmed cell death characterized by the excessive accumulation of copper within mitochondria [6,7,8]. Excess copper dysregulates mitochondrial functions by reducing the membrane potential, inhibiting ATP synthesis, and compromising electron transport chain (ETC) activity [9]. These disruptions lead to impaired cellular bioenergetics and an accumulation of reactive oxygen species (ROS), resulting in substantial damage to lipids, proteins, and DNA, ultimately culminating in cell death [10]. Compared to normal cells, cancer cells are particularly reliant on copper, given their rapid proliferation and requirements for antioxidant defense due to their high metabolic demands. Liver, colorectal, lung, pancreas, and breast cancer tissues also have higher copper concentrations than normal tissues [11,12]. Such elevated copper levels have been found to be associated with the induction of malignant transformation in hepatocellular carcinoma and pancreatic ductal adenocarcinoma, indicating a strong link between copper homeostasis dysregulation and cancer occurrence [13,14]. Overloading copper has been studied as a cancer therapeutic strategy for inducing mitochondrial dysfunction, which leads to proteotoxic stress [8,15]. Notably, protein aggregation is a major characteristic of cuproptosis, with copper-induced disturbances in protein folding leading to the oligomerization of lipoylated dihydrolipoamide S-acetyltransferase (DLAT), triggering a proteotoxic stress response (PSR) [8].

Heat shock factor 1 (HSF1) is a central regulator of the evolutionarily conserved PSR, acting as the master regulator of stress responses [16,17]. In response to proteotoxic stressors, such as heat shock or exposure to heavy metals like copper, HSF1 undergoes activation and nuclear translocation to initiate the transcriptional activation of genes encoding heat shock proteins (HSPs) [18]. HSPs are essential chaperones that facilitate the refolding of misfolded or aggregated proteins, thereby preventing their accumulation and promoting their degradation through the proteasomal or autophagy pathways [19]. Dysregulations in these processes can lead to the accumulation of misfolded or aggregated proteins, disrupting intracellular ion homeostasis and interfering with cellular functions [20]. Under normal physiological conditions, HSF1 not only monitors intracellular protein folding and quality control processes to ensure the proper functionality of the cellular proteome but also maintains proteome homeostasis (proteostasis) by directly interacting with misfolded proteins [21,22,23]. Protein aggregation is a hallmark of various neurodegenerative diseases, and amyloidogenesis refers to the generation of amyloid fibers during the process of proteotoxic stress [20,22]. As a part of the PSR, HSF1 physically interacts with these amyloid fibers in maintaining proteostasis [21,22,24]. Thus, HSF1 surveils the cellular proteome for aberrant protein folding events and coordinates the expression of molecular chaperones to ensure proper protein folding and prevent the accumulation of misfolded or aggregated proteins [21,22,24]. Through its role in the continuous surveillance and maintenance of proteostasis, HSF1 contributes to promoting cellular resilience against various stressors and helps preserve cellular homeostasis.

In this study, we investigate the pivotal role of HSF1 in maintaining cellular homeostasis by regulating protein folding and quality control mechanisms, particularly in the context of copper-induced toxicity and protein aggregation. Through its coordination of the cellular response to proteotoxic stress induced by copper exposure, HSF1 ensures the proper folding of proteins and contributes to overall cellular health and resilience against various stressors. Additionally, we explore the specific involvement of HSF1 in mitigating DLAT aggregation, a key event in copper-induced cellular damage. Understanding the mechanisms underlying HSF1-mediated proteostasis and its role in counteracting copper-induced toxicity and protein aggregation has significant implications for elucidating cellular pathologies and developing targeted therapeutic interventions for diseases associated with protein misfolding and aggregation.

## 2. Results

### 2.1. Copper Induces a Proteotoxic Stress Response and Activates HSF1 

**Figure 1:**
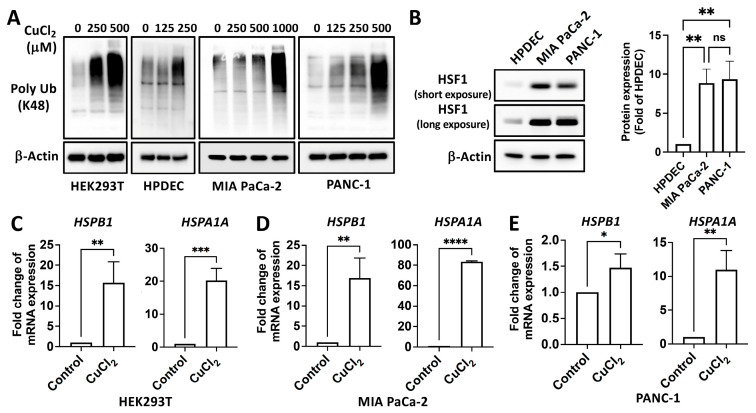
Copper induces a proteotoxic stress response. (**A**) HEK293T, HPDEC, MIA PaCa-2, and PANC-1 cells were treated with the indicated doses of CuCl_2_ for 24 h. (**B**) HSF1 expression in HPDEC, MIA PaCa-2, and PANC-1 cells. (**C**) HEK293T cells treated with 0.5 mM CuCl_2_ for 8 h. (**D**) MIA PaCa-2 cells treated with 1 mM CuCl_2_ for 8 h. (**E**) PANC-1 cells treated with 0.5 mM CuCl_2_ for 8 h. Protein detection using Western blotting (**A**,**B**), and detection of mRNA levels using RT-qPCR (**C**,**D**). Poly Ub: Poly-ubiquitination. *N* = 3. ns, not significant, *p* > 0.05, *, *p* < 0.05, **, *p* < 0.01, ***, *p* < 0.001, ****, *p* < 0.0001.

**Figure 2:**
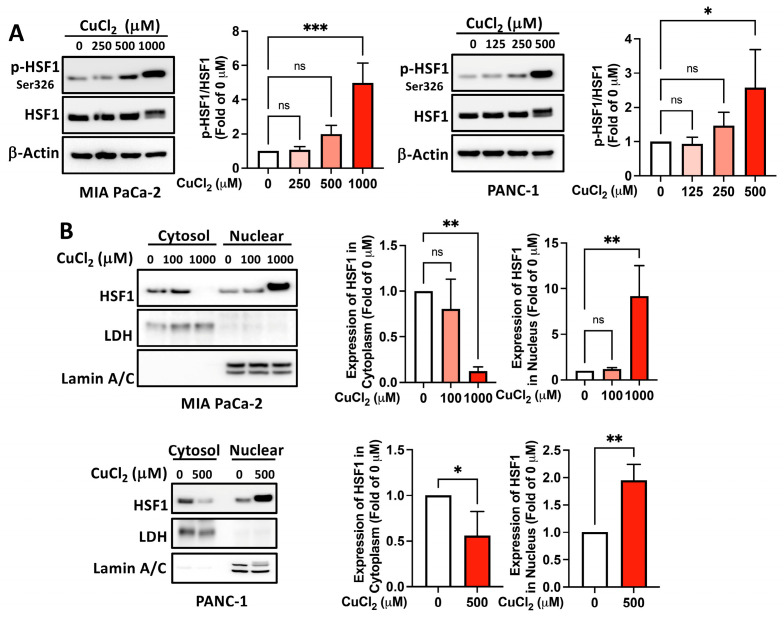
Copper treatment induces phosphorylation and nuclear translocation of HSF1. MIA PaCa-2 or PANC-1 cells were treated with indicated doses of CuCl_2_ for 12 h. (**A**) Phosphorylation of HSF1 (p-HSF1) at Ser326 levels and HSF1 expression in whole cell lysate was accessed by Western blotting. (**B**) Cytosol and nuclear fractions were separated as described in the Materials and Method sections, and the subcellular distribution of HSF1 was examined by Western blotting. All Western blot data were quantified and presented as bar graphs. Representative images from *N* = 3. ns, not significant, *p* > 0.05, *, *p* < 0.05, **, *p* < 0.01, ***, *p* < 0.001.

To examine the role of copper in the PSR, we treated human embryonic kidney (HEK) 293T, immortalized human pancreatic ductal epithelial cells (HPDEC), human pancreatic ductal adenocarcinoma cells (PDAC), including MIA PaCa-2, and PANC-1 cell lines with various doses of copper chloride (CuCl_2_). With the treatment of a higher concentration of copper as a model, copper-induced global protein polyubiquitination (Figure 1A), implying the occurrence of a PSR, as well as the expression of downstream *HSP* mRNA (Figure 1C–E), indicating the activation of HSF1 within these cell models, as similarly occurs during a heat shock response. PDAC exhibited high HSF1 expression, suggesting tolerance to copper-induced toxicity (Figure 1B). HSF1-mediated PSR is regulated through HSF1 activation, characterized by phosphorylation of HSF1 at Ser326, migration shift, nuclear translocation, and DNA binding. To confirm the copper-induced activation of HSF1, we treated MIA PaCa-2 and PANC-1 cells with increasing doses of CuCl_2_. In both MIA PaCa-2 and PANC-1 cell lines, copper exposure led to a dose-dependent rise in HSF1 phosphorylation at Ser326. Additionally, HSF1 nuclear translocation was observed, supporting copper-induced PSR (Figure 2A,B). These results demonstrate that copper treatment induces the activation of HSF1. 

### 2.2. HSF1 Reduces Copper Accumulation 

**Figure 3:**
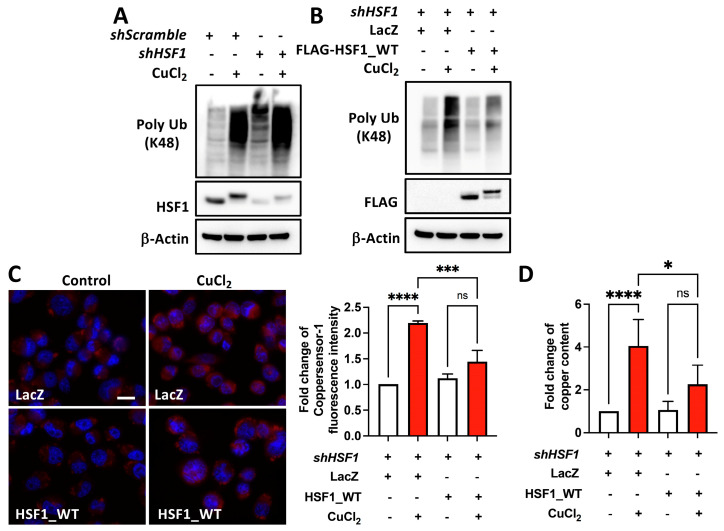
HSF1 reduces copper accumulation. (**A**) HSF1-deficient or (**B**) HSF1-overexpressing (FLAG-HSF1_WT) HSF1-deficient MIA PaCa-2 cells treated with 1 mM CuCl_2_ for 12 h, with detection of poly Ub, HSF1, and FLAG-HSF1_WT protein levels using Western blotting. Representative images from *N* = 3. Copper content measurement using Coppersensor-1 and detected by fluorescence microscopy (**C**, left), and the Coppersonsor-1 fluorescence intensity was quantified using Image J software (Version 2.3.0) from the images (**C**, right) or ELISA (**D**). Scale bar: 20 μm. *N* = 3–5. ns, not significant, *p* > 0.05, *, *p* < 0.05, ***, *p* < 0.001, ****, *p* < 0.0001.

We next examined the HSF1 expression levels in copper-induced protein polyubiquitination. The knockdown or overexpression of HSF1 modulates copper-induced global protein polyubiquitination in MIA PaCa-2 cells (Figure 3A,B). After confirming the activation of HSF1 upon copper addition, we further examined whether HSF1 had a role in copper accumulation using MIA PaCa-2 cells with endogenous HSF1 knockdown, without or with ectopic expression of HSF1. We treated these cells with CuCl_2_ and found that cells overexpressing HSF1 accumulate lower levels of copper compared to cells overexpressing LacZ (Figure 3C,D). Hence, HSF1 plays an important role in copper homeostasis.

### 2.3. HSF1 Plays a Crucial Role in Copper-Reduced Cell Viability 

**Figure 4:**
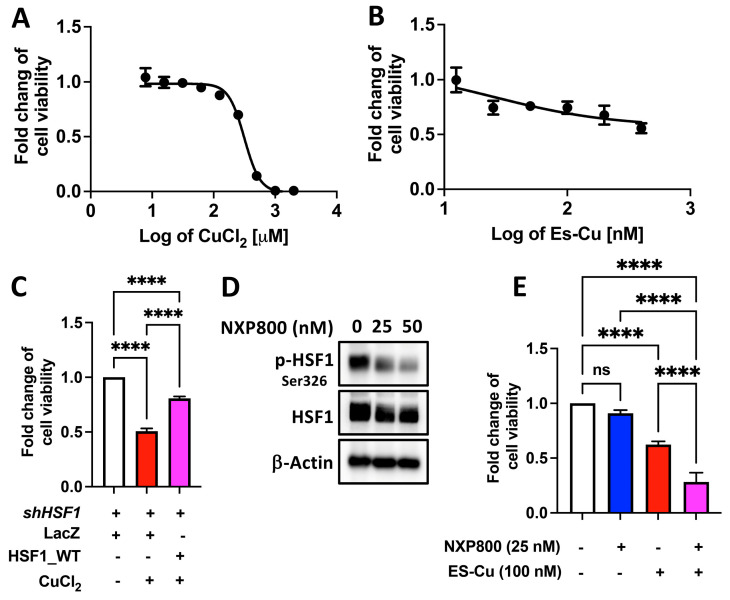
HSF1 protects cells from copper-induced toxicity. MIA PaCa-2 cells were treated with various doses of (**A**) CuCl_2_ or (**B**) ES-Cu for 72 h. *N* = 4. (**C**) HSF1-deficient MIA PaCa-2 cells without or with HSF1 overexpression treated with 0.5 mM CuCl_2_ for 72 h. *N* = 4. (**D**) MIA PaCa-2 cells treated with the indicated dose of NXP800 for 24 h. Protein levels were detected by using Western blotting. *N* = 1. (**E**) MIA PaCa-2 cells treated with 25 nM NXP800, 100 μM ES-Cu, or a combination for 72 h. Detection of cell viability using CellTiter-Blue^®^ Cell Viability Assay. Signals were measured using an ELISA plate reader. *N* = 4. ns, not significant, ****, *p* < 0.0001.

We investigated the cell death induced by CuCl_2_ and Elesclomol-Cu(II) (ES-Cu) (Figure 4A,B). The IC50 of the CuCl_2_ is 447.4 μM, and it is unmeasurable for ES-Cu, which might be due to the resistance [25]. Overexpression of HSF1 attenuates the CuCl_2_-induced reduction in cell viability (Figure 4C). Furthermore, to determine the involvement of HSF1 in the copper-induced reduction in cell viability, we treated cells with NXP800, a small-molecule HSF1 inhibitor currently in phase Ib clinical trials [26,27], which reduced the phosphorylation of HSF1 at Ser326 (Figure 4D) and observed reduced cell viability upon the addition of ES-Cu (Figure 4E). These data suggest that HSF1 protects cells by suppressing copper-reduced cell viability.

### 2.4. HSF1 Mitigates Copper-Induced Protein Aggregation 

**Figure 5:**
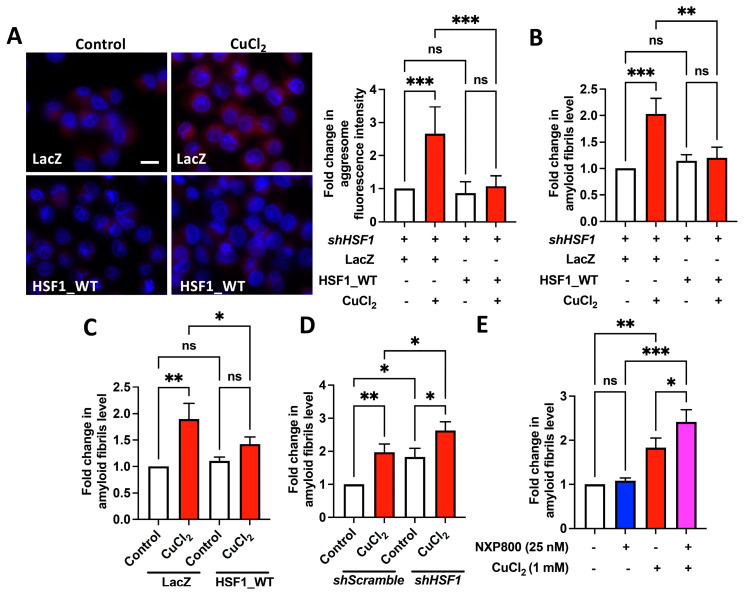
HSF1 ameliorates CuCl_2_-induced amyloid fibril formation. (**A**,**B**) HSF1-overexpressing (HSF1_WT) HSF1-deficient MIA PaCa-2 cells were treated with 1 mM CuCl_2_ for 24 h. (**C**) LacZ- or HSF1_WT-overexpressed HEK293T cells were treated with 0.5 mM CuCl_2_ for 24 h. (**D**) HSF1-deficient (shHSF1) MIA PaCa-2 treated with 1 mM CuCl_2_ for 24 h. (**E**) MIA PaCa-2 cells were treated with 25 nM NXP800 for 2 h, followed by 1 mM CuCl_2_ for 24 h. Aggresomes were imaged using a fluorescence microscope (**A**, left), and the measured aggresome fluorescence intensity was quantified using Image J software (Version 2.3.0) (**A**, right). Scale bar: 20 μm. Amyloid fibrils detection using an anti-amyloid fibril (anti-OC) antibody, with detection of signals using ELISA (**B**–**E**). *N* = 3. ns, not significant, *p* > 0.05, *, *p* < 0.05, **, *p* < 0.01, ***, *p* < 0.001.

As the increase in global protein polyubiquitination of cells treated with copper suggests the accumulation of misfolded or aggregated proteins (Figure 1), we therefore characterized protein aggregation. Aggresome accumulation and amyloid fibril formation are common responses to both cellular stress and the presence of misfolded or aggregated proteins and can therefore serve as biomarkers of protein aggregation and cellular dysfunction [28,29]. As expected, overexpressing HSF1 reduces copper-induced aggresome accumulation and amyloid fibril formation (Figure 5A–C). Both HSF1 knockdown and NXP800 treatment enhanced the accumulation of copper-induced amyloid fibrils (Figure 5D,E).

### 2.5. HSF1 Prevents the Transition of DLAT into the Insoluble Fraction Under Copper Treatment

**Figure 6:**
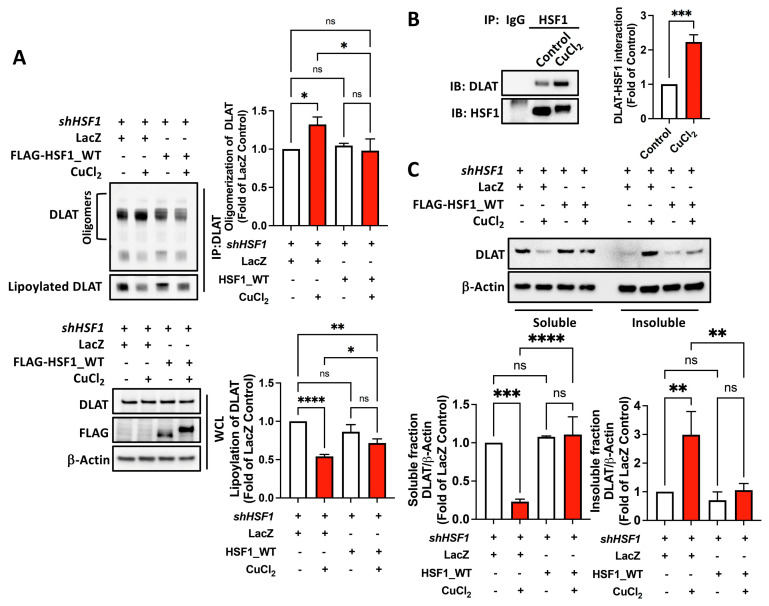
HSF1 suppresses DLAT from becoming insoluble under copper treatment. (**A**) HSF1-overexpressing HSF1-deficient MIA PaCa-2 cells treated with 1 mM CuCl_2_ for 12 h, with posttranslational modification of DLAT detected using a nonreducing gel (upper) and protein from whole cell lysate (WCL) detected using Western blotting (lower). (**B**) DLAT–HSF1 interaction was detected using immunoprecipitation (IP) and immunoblotting (IB). (**C**) HSF1-overexpressing HSF1-deficient MIA PaCa-2 cells treated with 1 mM CuCl_2_ for 12 h with isolation of the detergent-soluble (soluble) and detergent-insoluble (insoluble) fractions. *N* = 3. ns, not significant, *p* > 0.05, *, *p* < 0.05, **, *p* < 0.01, ***, *p* < 0.001, ****, and *p* < 0.0001.

Copper-induced cell death processes are known to include a reduction in DLAT lipoylation and the oligomerization of lipoylated proteins associated with the TCA cycle [8]. Accordingly, we observed a decrease in DLAT lipoylation and an increase in DLAT oligomerization in cells treated with copper, whereas overexpression of HSF1 reversed this effect (Figure 6A). Additionally, copper induced a PSR, resulting in the enhancement of the interaction between HSF1 and DLAT (Figure 6B). Previous studies have indicated that protein aggregation decreases protein solubility, detectable through an increase in the detergent-insoluble fraction [22,24]. We observed that copper induced a decrease in the levels of detergent-soluble DLAT and an increase in those of detergent-insoluble DLAT, with both trends attenuated by HSF1 overexpression (Figure 6C). These findings suggest a role for HSF1 in mitigating copper-induced DLAT aggregation.

## 3. Discussion

Copper is essential for cellular functions involving enzymatic reactions and electron transport across mammalian cells [1,2]. Excessive levels of intracellular copper ions induce cellular toxicity and the PSR (Figure 8) [8]. Cells initiate PSR mechanisms that induce HSF1-HSP chaperone activity to repair abnormalities and maintain protein equilibrium [18,19]. Our results reveal that overloaded copper induces HSP expression, HSF1 phosphorylation, and HSF1 nuclear translocation in human PDAC cells and demonstrate, for the first time, the role of HSF1 in mitigating copper overload-induced copper accumulation, protein ubiquitination, protein aggregation, and loss of cell viability in human PDAC cells. Although copper overload results in cytoplasmic HSF1 aggregation in zebrafish embryonic hematopoietic progenitor cells [30], in our study, HSF1 overexpression prevented, whereas HSF1 inhibition promoted, overloaded copper- or ES-Cu-reduced cell viability. HSF1 is a pro-oncogenic factor that is highly expressed for tumor survival in most cancer types, including pancreatic cancer. We observe that compared to HPDEC cells, much higher copper doses are required to induce protein ubiquitination in PDAC. The observed requirement for a higher copper dose to induce global protein ubiquitination in MIA PaCa-2 cells could be attributed to the cellular heterogeneity associated with KRAS mutations, which may influence the copper sensitivity and overall cellular protein ubiquitination response [31,32]. These results reinforce our understanding of HSF1′s protective role in tumor resistance to toxic copper levels. 

**Figure 7:**
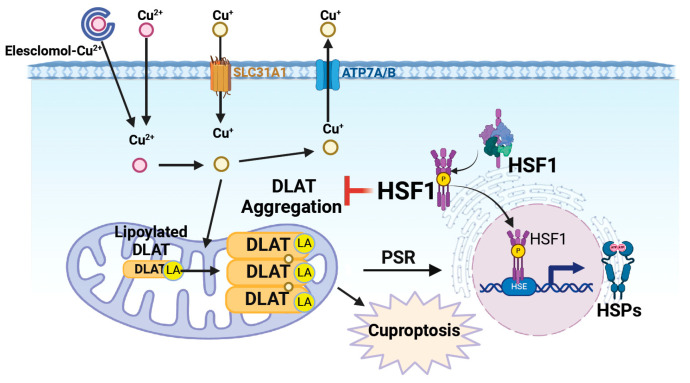
Role of HSF1 in copper-induced toxicity. Lipoylation allows DLAT to facilitate the transfer of acetyl groups during metabolic processes, which is essential for the proper functioning of the TCA cycle. Copper induces DLAT oligomerization and initiates PSR. HSF1 inhibits copper-induced insoluble fraction of DLAT.

The use of chelators or ionophores is a common strategy to modulate cellular copper levels [33]. Chelators directly bind and sequester metal ions, whereas ionophores bind metal ions on one side of the cellular membrane, traverse the cellular membrane, and then release metal ions on the other side of the cellular membrane, typically leading to increased intracellular metal ion concentrations [34,35]. One of the more novel ionophores is Elesclomol, which has shown significant toxicity against many types of malignant cells, such as cancer stem cells, metastatic tumor cells, therapy-resistant tumor cells, and tumors with suppressed glycolysis, by disrupting copper homeostasis and inducing cuproptosis, and it is undergoing clinical trials [36,37,38,39]. Copper homeostasis is mediated by several transporters, most notably the importer copper transporter (CTR1, SLC31A1) and the exporter ATPase copper transporting alpha/beta (ATP7A/B) transporters [2,11,40]. ES-Cu has been shown to reduce ATP7A/B expression, thereby resulting in intracellular copper accumulation (Figure 7). The addition of Elesclomol with copper increases the death of colorectal cancer cells, highlighting the importance of copper in the processes of this type of cancer [41,42]. In our study, although further study is required to investigate the detailed mechanism, we find that overexpression of HSF1 eliminates the copper-induced copper accumulation, implying the HSF1′s role in intracellular copper homeostasis.

Insufficient machinery for repairing misfolded proteins leads to their aggregation and the subsequent formation of amyloid fibrils, which are highly toxic to cells [24]. Several pathological conditions are predominately characterized by this progression of protein aggregation and amyloid fibril formation, among which the most notable is Alzheimer’s Disease (AD), where symptomatic progression is directly tied to amyloid beta accumulation in neuronal cells [43,44]. HSF1 expression is decreased in AD patients, and overexpression of the active form of HSF1 in AD animal models reverses cognitive defects [43,44,45]. HSF1 directly binds to and prevents Aβ_1–42_ aggregation, indicating that HSF1 functions as an anti-amyloid factor [21,22]. In cancer cells, inhibition of MEK signaling inactivates HSF1 and induces protein destabilization, aggregation, and amyloidogenesis, which induces cell toxicity, thereby contributing to the anti-tumor effect [24,46]. Copper treatment has been shown to induce cytotoxicity and accompany the PSR [6,8,47]. Short-term treatment with high concentrations (>1000 μM) of CuCl_2_ has been used to investigate cellular toxicity in human neuroblastoma, breast cancer, colorectal adenocarcinoma, and keratinocyte cell lines [41,48,49,50]. Copper accumulation in cells can cause proteotoxic stress, as we demonstrated, which is known to transiently activate HSF1, whereas overexpression of HSF1 reduces copper accumulation. This regulation could represent a negative feedback loop where excess copper activates HSF1, which in turn activates pathways that mitigate further copper accumulation, preventing cellular damage. Further mechanistic studies would be needed to confirm this feedback system and identify the specific downstream targets of HSF1 involved in copper homeostasis, which is the project we are working on. We find that exposure of HEK293T or PDAC cells to copper or ES-Cu induces the formation of amyloid fibrils and that overexpression of HSF1 significantly reduces copper-induced aggresome accumulation and amyloid fibril formation, indicating a cytoprotective role for HSF1 in combating stress. Copper-induced toxicity may involve other distinct programmed cell death signals, but further study is required to elucidate the mechanism of sensitivity and their response to the copper chelators. The role HSF1 plays in the reduction in protein aggregation in several conditions is further evidence of its role in broader proteasomal stability. Further understanding of this pathway can thus be applied and studied across several diseases. There are several clinical trials investigating HSF1 as a therapeutic target, such as a phase Ib study (NCT05226507) investigating NXP800 in ovarian carcinoma [26,27]. Our findings reveal that HSF1 protects cells from copper-reduced cell viability; thus, combining HSF1 inhibition with copper treatment represents a novel therapeutic strategy for cancer treatment.

Copper overloading disturbs the usual folding and structure of cellular proteins, resulting in the oligomerization of lipoylated DLAT, which is necessary for cuproptosis [8,51,52]. The oligomerized DLAT forms protein aggregates and triggers a PSR [8]. This PSR plays dual roles in either maintaining cellular integrity or, when unsuccessful, serving as a marker indicating the beginning of cellular damage and programmed cell death [53,54,55,56]. In our study, treatment with copper leads to uncontrolled DLAT oligomerization and protein aggregation, whereas overexpression of HSF1 reverses copper-induced oligomerization of DLAT but partially reverses copper-reduced DLAT lipoylation. This reduction in the DLAT oligomerization explains the reduced copper-induced DLAT aggregation under HSF1 overexpression. These findings suggest that HSF1 plays a role in regulating DLAT posttranslational modification, including lipoylation and oligomerization. Copper is involved in electron transport by facilitating the movement of electrons along the electron transporter chain, which is crucial for ATP synthesis [8,9]. Electrons are transferred via a series of protein complexes, including ferredoxin 1, a member of the iron–sulfur (Fe–S) cluster protein family [52,57,58]. These proteins act as essential electron donors in various cellular processes, ultimately leading to ATP production. Additionally, Fe–S cluster-related genes are involved in the lipoic acid biosynthesis pathway, which promotes DLAT lipoylation [52,57,58]. Our results show that overexpression of HSF1 partially restores the copper-reduced DLAT lipoylation, implying that HSF1 might play a regulatory role in the expression or function of Fe–S cluster-related proteins. However, the detailed mechanism remains to be elucidated. Interestingly, we find that HSF1 directly interacts with DLAT under copper treatment and HSF1 overexpression suppresses copper-induced DLAT aggregation. HSF1 has been reported to co-purify with isolated mitochondria [59]. It also colocalizes in mitochondria under non-stressed conditions, with its presence increasing under heat shock conditions in yeast cells [60]. Additionally, HSF1 antagonizes amyloidogenesis in mitochondria by physically interacting with β amyloid to prevent β amyloid aggregation through both transcriptional and non-canonical roles [22]. Overexpression of HSF1 protects cells from copper-induced cytotoxicity by preventing the formation of insoluble DLAT aggregates. This mechanism explains how HSF1 mitigates copper-induced DLAT insolubility and associated cellular toxicity. However, HSF1 likely prevents DLAT insoluble fraction formation indirectly through the induction of chaperone proteins, the ubiquitin-proteasome system, and autophagy pathways [22,61]. The direct mechanism by which HSF1 prevents amyloidogenesis and protein aggregation remains elusive. Hence, further study needs to be conducted to investigate the role of HSF1 in copper-induced protein insolubility. 

Our work highlights the complex relationship between copper and cellular stress responses, shedding light on metal-related diseases. We demonstrate how HSF1 mitigates copper-induced cellular damage, suggesting broader implications for protein aggregation-mediated disorders. Further studies are needed to elucidate the mechanism by which HSF1 regulates metal ion transporters and the Fe–S cluster protein expression in multiple cancer cell types, which is the project we are now working on. Through future research in this field, there is potential for discovering more therapeutic targets and developing improved tactics for treating copper-related toxicity responses.

## 4. Materials and Methods

### 4.1. Cells and Reagents

HEK293T cells and pancreatic cancer cell lines MIA PaCa-2 and PANC-1 were procured from the American Type Culture Collection (ATCC; Manassas, VA, USA). HPDEC was purchased from AddexBio (San Diego, CA, USA). HSF1 inhibitors NXP800 (HY-145927), Elesclomol-Cu(II) (HY-156376), and Coppersensor-1 (HY-141511) were obtained from MedChemExpress LLC (MCE; Monmouth Junction, NJ, USA). Copper(II) chloride (AA1245718) and Hoechst 33342 (H1399), were sourced from Thermo Fisher Scientific (Waltham, MA, USA). CellTiter-Blue^®^ Cell Viability Assay (G8080) was purchased from Promega (Madison, WI, USA), and the PROTEOSTAT^®^ Aggresome Detection Kit (ENZ-51035-K100) was acquired from Enzo Life Sciences (Long Island, NY, USA). Poly-L-lysine (P4707) was purchased from Sigma-Aldrich (St. Louis, MO, USA). Additionally, RT Master Mix for qPCR II (HY-K0510A) and SYBR Green qPCR Master Mix (Low ROX) (HY-K0522) were obtained from MCE.

### 4.2. Cell Culture 

HEK293T and PANC-1 cells were grown in high-glucose Dulbecco’s minimal essential medium (DMEM; Gibco, Palo Alto, CA, USA) supplemented with 10% fetal bovine serum (Hyclone Laboratories, Logan, UT, USA), 1% penicillin/streptomycin (Gibco), and 1% sodium pyruvate (Gibco). MIA PaCa-2 cells were grown in high-glucose DMEM (Gibco) supplemented with 1.25% horse serum (Gibco), 10% fetal bovine serum, and 1% sodium pyruvate. HPDEC cells were grown in keratinocyte serum-free medium supplemented with bovine pituitary extract and human recombinant epidermal growth factor (Invitrogen, Waltham, MA, USA) [62]. Cells were incubated in a humidified environment with 5% carbon dioxide at a constant temperature of 37 °C until 70% confluence was reached. 

### 4.3. Immunoblotting and Immunoprecipitation

Whole-cell protein lysates were prepared using cold lysis buffer containing 100 mM NaCl, 30 mM Tris-HCl pH 7.6, 1% Triton X-100, 20 mM sodium fluoride, 1 mM EDTA, 1 mM sodium orthovanadate, and 1× Halt protease inhibitor cocktail [49,50]. Nucleus and cytosol extraction were conducted using NE-PER™ Nuclear and Cytoplasmic Extraction Reagents (Thermo Fisher Scientific) and following the manufacturer’s instructions [50]. Samples were derived from the same experiment or parallel experiments, and gels/blots were processed in parallel. Protein quantification was performed using the Pierce BCA protein assay kit from Thermo Fisher Scientific (#23227). Total proteins (25–40 μg) were resolved on 8–15% SDS-PAGE gels before being transferred to nitrocellulose membranes. The membranes were blocked with a blocking solution (5% nonfat milk in 1× Tris buffered saline with Tween-20; TBS-T) for 1 h at room temperature and then incubated overnight at 4 °C with primary antibodies diluted at 1:1000 in the blocking buffer. After washing with 1× TBS-T buffer three times, the membranes were incubated with peroxidase-conjugated secondary antibodies (diluted at 1:1000 in the blocking solution) at room temperature for one hour. Signals were detected using SuperSignal^TM^ West Pico PLUS chemiluminescent substrate (Thermo Fisher Scientific) and analyzed using ImageJ software (Version 2.3.0, National Institutes of Health; NIH, Bethesda, MD, USA). 

For immunoprecipitation (IP), 1 mg of whole cell lysate was preincubated with Protein G MagBeads to block nonspecific binding. Subsequently, the cell lysates were incubated with anti-DLAT or HSF1 antibodies at 4 °C overnight. Normal rabbit IgG (Cell Signaling Technology, Beverly, MA, USA) was utilized as a negative control. Protein G MagBeads (MCE) were preincubated with 1% BSA at 4 °C for 1 h, and then the washed Protein G MagBeads were used to precipitate the primary antibodies. After washing with lysis buffer three times, the beads were boiled in 1× loading buffer with β-mercaptoethanol for 5 min before loading onto SDS-PAGE. For posttranslational modification of DLAT, the beads were boiled in 1× loading buffer without β-mercaptoethanol for 5 min before loading onto nonreducing SDS-PAGE. 

### 4.4. Antibodies

The antibodies used in this study were sourced from various suppliers: HSF1 (D3L8I) Rabbit mAb (#12972) and Lamin A/C Antibody (#2032) were obtained from Cell Signaling Technology, DYKDDDDK Tag (D6W5B) Rabbit mAb (#14793) and DLAT (4A4-B6-C10) Mouse mAb (#123620) were also acquired from Cell Signaling Technology, β-Actin Mouse mAb (AC004) was procured from ABclonal, Inc. (Woburn, MA, USA), Anti-Ubiquitin (linkage-specific K48) antibody [EP8589] (ab140601) and Rabbit anti-LDH monoclonal antibody [EP1563Y] (Cat# 1980-1) were purchased from Abcam (Waltham, MA, USA), Anti-Phospho-HSF1-S326 antibody produced in rabbit (SAB5701906) was acquired from Sigma-Aldrich and in rabbit (YA894) was from MCE, and Anti-Amyloid Antibody (SPC-507) (OC) Rabbit Polyclonal antibody was obtained from StressMarq Biosciences Inc. (Victoria, BC, Canada).

### 4.5. Quantitative RT-PCR

After cell harvesting, RNA was extracted using STAT60 reagent (Tel-Test, Friendswood, TX, USA) and converted to cDNA using RT Master Mix for qPCR II (Bio-Rad; Hercules, CA, USA). Subsequently, quantitative reverse transcription polymerase chain reaction (qRT-PCR) analysis was performed using gene-specific primers and Luna^®^ Universal qPCR Master Mix (New England Biolabs, Inc., Ipswich, MA, USA) on a 7500 Real-Time PCR System (Thermo Fisher Scientific). The synthesized sequences of primers (Integrated DNA Technologies, Inc. Coralville, IA, USA) used for qPCR according to the OriGene primer pair (HP208499; OriGene Technologies, Inc., Rockville, MD, USA) are as follows: Human_*HSPA1A/HSP72*_Forward: 5′-ACCTTCGACGTGTCCATCCTGA-3′; Human_*HSPA1A/HSP72*_Reverse: 5′-TCCTCCACGAAGTGGTTCACCA-3′; Human_*HSPB1/HSP27*_Forward: 5′-GGACGAGCTGACGGTCAAG-3′; Human_*HSPB1/HSP27*_Reverse: 5′-AGCGTGTATTTCCGCGTGA-3′.

### 4.6. Plasmid Construction and HSF1 Overexpressed Rescue Cell

The plasmids utilized in this study, including VSVG and dVPR, were obtained from Addgene (Watertown, MA, USA). The plasmid containing the shRNA targeting HSF1 (GCAGGTTGTTCATAGTCAGAA) was acquired from Sigma-Aldrich (TRCN0000007480), while the Scramble control shRNA (CCTAAGGTTAAGTCGCCCTCG) was sourced from Addgene (#1864). Additionally, plasmids pLenti-LacZ and pLenti-HSF1_WT_FLAG were gifts generously provided by Dr. Chengkai Dai at the Mouse Cancer Genetics Program, National Cancer Institute (Frederick, MD, USA) and have been previously described [63,64].

Lentivirus was produced through the transduction of pLenti-LacZ, or pLenti-HSF1-WT-FLAG mixed with an optimal ratio of VSVG and dVPR into HEK293T cells using jetPRIME^®^ DNA and siRNA Transfection Reagent from PolyPlus Transfection (New York, NY, USA), as previously described [63,64]. Virus-containing medium was harvested 48 h post-transduction, and the titer was determined using the Lenti-X GoStix Plus kit (Cat. No. 631280, Takara Bio USA, Inc., San Jose, CA, USA) following the manufacturer’s instructions.

MIA PaCa-2 cells were infected with either shScramble or shHSF1 virus in the presence of 10 μg/mL polybrene (Sigma-Aldrich) for 48 h. Subsequently, cells were infected with pLenti-LacZ or pLenti-HSF1_WT_FLAG in the presence of 10 μg/mL polybrene for an additional 48 h. The levels of HSF1 and overexpressed HSF1_WT_FLAG were assessed using Western blotting.

### 4.7. Copper Content Measurement

The intracellular copper content was measured using Coppersensor-1 (MCE). Briefly, after the indicated treatment and washed with 1× phosphate-buffered saline (PBS), cells were incubated with 5 μM Coppersensor-1 in PBS at 37 °C to protect from light for 30 min. The copper content was measured by ELISA and normalized by cell viability. The nuclei were counterstained with Hoechst 33342 (Thermo Fisher Scientific). The Coppersensor-1-labeled cells were imaged using an Axio Observer 3 inverted fluorescence microscope (Carl Zeiss, Berlin, Germany), and Coppersonsor-1 fluorescence intensity was quantified using Image J software (Version 2.3.0) (NIH). 

### 4.8. Cell Viability Assay 

MIA PaCa-2 cell lines were seeded in 96-well plates at a density of 4,000 cells per well and treated with various drugs for the indicated times. Cell viability was assessed using the fluorometric resazurin reduction method (CellTiter-Blue^®^ Cell Viability Assay; Promega, Madison, WI, USA). Fluorescent signals were measured at excitation and emission wavelengths of 560 nm and 590 nm, respectively, using a SpectraMax^®^ iD5 Multi-Mode Microplate Reader (Molecular Devices, LLC., San Jose, CA, USA).

### 4.9. Aggresome Assay

Aggresomes were detected according to the manufacturer’s instructions. Briefly, cells were seeded on poly-L-lysine-coated glass slides. After treatment, cells were fixed with 4% paraformaldehyde for 30 min. Following aspiration of the paraformaldehyde, cells were washed with PBS and incubated with Permeabilizing Solution on ice for 30 min. After washing off the permeabilizing solution, cells were incubated with Dual Detection Reagent for 30 min at room temperature. Subsequently, the detection reagent was removed, and cells were washed with 1× PBS before counterstaining of nuclei with Hoechst 33342 (Thermo Fisher Scientific) and covered with a cover glass. Aggresomes and nuclei were visualized using an Axio Observer 3 inverted fluorescence microscope (Carl Zeiss), and aggresome fluorescence intensity was quantified using Image J software (Version 2.3.0) (NIH).

### 4.10. Fractionation of Cell Lysates to Separate Soluble and Insoluble Fractions

Detergent-soluble and -insoluble fractions were separated as previously described [22,24]. Briefly, cell lysates were prepared following treatment, with equal cell numbers collected. Lysis was performed using 1% Triton X-100 buffer with protease inhibitor, followed by centrifugation at 500× *g* for 5 min at 4 °C to obtain the soluble fraction (supernatant). The pellet containing insoluble and membrane-soluble fractions was subjected to DNase treatment to digest genomic DNA, followed by resuspension in 2% SDS buffer. After centrifugation, the supernatant was collected as the membrane-associated fraction, while the pellet was sonicated to break down the insoluble fraction for the anti-OC measurement or the Western blotting. 

### 4.11. Statistical Analysis 

All quantitated results are presented as mean ± SD from at least three independent replicates and evaluated using nonparametric tests. Image processing was performed using photograph imaging software Adobe Photoshop (Version: 25.12.0, Adobe Systems, Mountain View, CA, USA) and ImageJ (NIH). One-way ANOVA followed by Bonferroni test post-hoc analyses were employed for multiple testing, while for two-group comparisons, an unpaired *t*-test was utilized. Statistical significance was considered at *p* < 0.05 using GraphPad Prism 9.0 (GraphPad Software, Inc., San Diego, CA, USA).

## 5. Conclusions

Our findings clarify the molecular pathways responsible for the copper-induced reduction in cell viability and emphasize the protective function of HSF1 in reducing copper-induced DLAT aggregation. Copper treatment results in reduced DLAT lipoylation and increased DLAT oligomerization, aligning with the established mechanisms linked to copper-induced cell death. In addition, copper triggers the PSR, as shown through the observed changes in HSF1 and the increased interaction between HSF1 and DLAT. We demonstrate that increasing the levels of HSF1 reduces the formation of DLAT aggregates caused by copper, representing a new way to defend against copper-induced cell damage. These findings enhance our knowledge of the molecular pathways responsible for metal-induced cellular harm and provide suggestions for prospective treatment in terms of approaches to reduce metal toxicity in different clinical situations.

## Data Availability

The data that support the findings of this study are available from the corresponding author, upon request.
